# Two Fully Automated Web-Based Interventions for Risky Alcohol Use: Randomized Controlled Trial

**DOI:** 10.2196/jmir.2489

**Published:** 2013-06-06

**Authors:** Marc-Dennan Tensil, Benjamin Jonas, Evelin Strüber

**Affiliations:** ^1^Delphi-GesellschaftBerlinGermany; ^2^Federal Centre for Health Education (BZgA)CologneGermany

**Keywords:** alcohol abuse, binge drinking, Internet intervention, relapse prevention, randomized controlled trial

## Abstract

**Background:**

Excessive alcohol use is a widespread problem in many countries, especially among young people. To reach more people engaging in high-risk drinking behaviors, a number of online programs have been developed in recent years. Change Your Drinking is a German, diary-based, fully automated alcohol intervention. In 2010, a revised version of the program was developed. It is more strongly oriented to concepts of relapse prevention than the previous version, includes more feedback, and offers more possibilities to interact with the program. Moreover, the program duration was extended from 10 to 14 days.

**Objective:**

This paper examines whether the revised version of Change Your Drinking is more effective in reducing alcohol consumption than the original version.

**Methods:**

The effectiveness of both program versions was compared in a Web-based, open, randomized controlled trial with follow-up surveys 6 weeks and 3 months after registration. Participants were recruited online and were randomly assigned to either the original or the revised version of Change Your Drinking. The following self-assessed outcomes were used: alcohol use days, alcohol intake in grams, the occurrence of binge drinking and risky drinking (all referring to the past 7 days prior to each survey), and the number of alcohol-related problems.

**Results:**

A total of 595 participants were included in the trial. Follow-up rates were 58.0% after 6 weeks and 49.6% after 3 months. No significant group differences were found in any of the outcomes. However, the revised version was used by more participants (80.7%) than the original version (55.7%). A significant time effect was detected in all outcomes (alcohol use days: *P*=.002; alcohol intake in grams: *P*<.001; binge drinking: *P*<.001; alcohol-related problems: *P*=.004; risky drinking: *P*<.001).

**Conclusions:**

The duration and complexity of the program played a minor role in reducing alcohol consumption. However, differences in program usage between the versions suggest the revised version was more attractive to participants.

**Trial Registration:**

International Standard Randomized Controlled Trial Number (ISRCTN): 31586428; http://www.controlled-trials.com/ISRCTN31586428/ (Archived by WebCite at http://www.webcitation.org/6BFxApCUT)

## Introduction

Excessive alcohol use is associated with numerous health and social consequences and represents a major challenge for public health activities. It is estimated that approximately 4% of all deaths worldwide are due to the consumption of alcohol [1]. In Germany, more than 70,000 people die from alcohol use-related consequences each year [2].

Typically, people are introduced to alcohol at a young age, which makes the earliest possible prevention a priority. In Germany, the average age of the first binge drinking experience is between 15 and 16 years [3]. To a certain extent, experimenting with alcohol can be regarded as a normal part of adolescence [4,5]. However, regular binge drinking not only represents a direct health risk, it also can impair brain development substantially [6,7].

In Germany, the prevalence of excessive drinking is highest among young adults. Forty-two percent of those aged between 18 and 25 years engage in binge drinking at least once a month [3]; 40% of those aged between 18 to 20 years meet the criteria for problematic alcohol use as defined by the Alcohol Use Disorders Identification Test (AUDIT) [8]. Despite high levels of problematic alcohol use, few young people seek professional help. Globally, the World Health Organization (WHO) estimates that 78% of those in need for treatment due to alcohol abuse or dependence remain untreated although effective intervention methods are available [9].

Internet-based self-help programs can help to reduce this gap. Their advantages include easy accessibility and discreetness; therefore, they provide an appealing alternative especially to those who would abstain from face-to-face treatment because of fear of exposure or embarrassment. Meta-analyses show that online self-help programs have a rather small effect size, but because of their scalability they are a cost-effective way to reduce alcohol consumption in the population [10,11]. Online self-help alcohol interventions have been shown to be effective in the general adult population [12-15] as well as in college student samples [16-18], underage drinkers [19], and with young people in the workplace setting [20].

Since 2009, the German Federal Centre for Health Education, Bundeszentrale für gesundheitliche Aufklärung (BZgA), has offered Change Your Drinking, a free online self-help program for young adults with problematic alcohol use. Based on cognitive behavioral principles, it provides a consumption diary for 10 days and 1 brief tailored feedback at the end of this period. Statements from users previously collected in an unpublished Web survey, however, indicated a need for further and more elaborate feedbacks. Moreover, some users regarded the consumption diary as too simple. Based on this information, the intervention was revised and extended to 14 days, aiming to involve the users more deeply into the program and to provide them with a more sophisticated version of the consumption diary. These goals reflect recent findings according to which elaborated self-help alcohol interventions spanning over different sessions are more effective than single-session feedback interventions [12,13], and a higher degree of interactivity is associated with higher effect sizes [21]. Moreover, Web-based interventions with a higher intended usage were found to be more likely to be adhered to [22].

This paper examines whether the revised version of Change Your Drinking is more effective in reducing alcohol consumption than the original version. Outcomes were the alcohol use days, the total intake in grams, the occurrence of binge drinking (yes/no), and binge drinking (yes/no) for the past 7 days prior to each survey as well as the number of alcohol-related problems.

## Methods

### Study Design

A randomized controlled trial (RCT) was conducted to compare the 2 versions of Change Your Drinking with follow-up surveys 6 weeks and 3 months after registration. Participants were invited by email and reimbursed with a €10 shopping voucher for their participation. The study was approved by the ethics committee of the Department of Applied Human Sciences at the University of Magdeburg-Stendal (Ref 4973-15) and was registered with Current Controlled Trials (ISRCTN: 31586428).

The intervention and the study were purely Web-based on the addiction prevention website Drugcom [23] and the alcohol prevention website Kenn dein Limit (Know Your Limit) [24] both run by the BZgA. Recruitment of participants started in December 2010 and ended in March 2012. All users of the freely accessible Check Your Drinking self-assessment were invited for the study if they met the eligibility criteria described subsequently. The results of the self-assessment were used both for the Change Your Drinking program and as baseline data for the trial.

If the eligibility criteria (see Study Criteria) were met, users were informed about the study. A portable document format (PDF) file containing all relevant study details was offered for download (see Multimedia Appendix 1). Users who were willing to participate were then asked to register and to provide their informed consent by clicking an “I agree to participate” button. After successfully confirming their email addresses, participants were randomly assigned either to the original (version 1) or the revised program (version 2) by random number generator software. Researchers could not influence nor predict the randomization result. The participants were blind to the results of the randomization because they only received detailed information about the program version they were allocated to.

Those who opted not to participate in the study or who did not meet the eligibility criteria had full access to the original version of the program and were not included in any follow-up surveys.

### Measures

Trial data were collected via self-assessment in the baseline survey, as well as 6 weeks and 3 months afterwards. The past 7 days were used as reference period for alcohol consumption. In order to quantify the alcohol intake, participants were first asked to indicate their number of alcohol use days in the previous 7 days. Afterwards, they were requested to specify the number and type of alcoholic beverages consumed on each drinking day. Using these details, the amount of pure alcohol and the number of standard glasses (SG) per day was calculated. According to the BZgA, 1 SG in Germany corresponds to 10 grams of pure alcohol [25]. If 5 or more SG were consumed on any of the previous 7 days, this was classified as binge drinking.

Another outcome was risky consumption in the past 7 days, as defined by the following factors: (1) an average of more than 24 or 12 g (for males and females, respectively) of pure alcohol per day (these are the tolerable upper alcohol intake levels for males and females in Germany according to Burger and colleagues [26]), or (2) more than 5 days of consumption, or (3) at least 1 incident of binge drinking in the reference period. The definition of risky alcohol use has been derived from the guidelines for low-risk consumption as defined by the BZgA [27].

Alcohol-related problems were measured by a German version of McGee and Kypri’s [28] Alcohol Problems Scale (APS). The scale consists of 14 items describing negative consequences of alcohol consumption, such as vomiting, unprotected sexual intercourse, or blackouts, during the last 30 days. Each item is to be answered with yes, no, or “no answer.”

The AUDIT was used as part of the baseline survey to test for the study criteria. A cut-off of 8 points as suggested by Babor et al [29] was used to define risky alcohol consumption. As a measure of the program usage we tracked the diary usage (used at least once: yes/no).

### Study Criteria

To be invited for the study, participants had to be Internet literate, at least 18 years old, and had either reached the cut-off of 8 points in AUDIT or have consumed more than 24/12 g (male/female) of pure alcohol per day on average in the past week. We did not use stricter study criteria (eg, exclusion of individuals currently in other treatment) because we wanted to increase the generalizability of the results to the regular users of Change Your Drinking.

### Interventions

Change Your Drinking is an Internet-based self-help program based entirely on automated tailored feedbacks. A comparison of both versions of the intervention is displayed in Table 1. Exemplary feedbacks of each version in German language are included in Multimedia Appendix 2.

**Table 1:** Comparison of both Change Your Drinking versions.

Characteristics	Version 1	Version 2
Aim	No alcohol use or low-risk use^a^	No alcohol use or low-risk use^a^
Duration	10 days	14 days
Interventions	Detailed tailored feedback at baseline with advice regarding the participant’s alcohol use (Check Your Drinking)	Detailed tailored feedback at baseline with advice regarding the participant’s alcohol use (Check Your Drinking)
	General information on control strategies	General information on control strategies
	10-day alcohol use diary	14-day alcohol use diary including a tool to help developing control strategies
		Daily: Short tailored feedback on the individual’s alcohol use and graphical display of alcohol use
		On day 7: Detailed tailored feedback on one’s alcohol use and tips on how to cope with risk situations
	On day 10: Tailored feedback on the individual alcohol consumption	On day 14: New detailed tailored feedback on one’s alcohol use and tips on how to cope with risk situations
		Advice to reflect one’s reasons for reducing or abstaining from alcohol
		Advice to reward oneself for achieving the personal goal

^a^Low-risk use was defined as (1) no more than 24/12 g (male/female) of pure alcohol per day [26], (2) no more than 5 alcohol use days, and (3) no incidents of binge drinking in past 7 days [25].

#### Original Version (Version 1)

Change Your Drinking is based on solution-focused brief intervention and methods of cognitive behavioral therapy. Berg and Miller’s [30] solution-focused treatment approach concentrates on achieving concrete behavioral goals within relatively narrow timeframes.

The first step in the program is a self-assessment tool (Check Your Drinking) which provides users with tailored feedback on their drinking behavior. If indicated, users are recommended to participate in the Change Your Drinking program.

At the beginning, participants of Change Your Drinking are to choose a use-related goal which must fall within the limits for low-risk consumption (for a definition of low-risk use see Table 1). Afterwards, the participants are given access to an online diary to keep track of their alcohol use over the next 10 days. Static information on alcohol control strategies is also given. The program aims to develop self-awareness and self-regulatory skills [31,32].

On the tenth day, participants receive feedback based on their current alcohol use and on how well the use-related goal was met. The feedback is based on Miller and Rollnick’s principles of Motivational Interviewing [33].

#### Revised Version (Version 2)

To promote confrontation with one’s own consumption pattern, the original version of Change Your Drinking was revised and supplemented with new modules. Based on the principles of relapse prevention [34,35], participants are now asked daily to confront their risk situations and to develop or refine control strategies. Short and motivating feedbacks are provided to reinforce the reflection of one’s own alcohol use.

**Figure 1:**
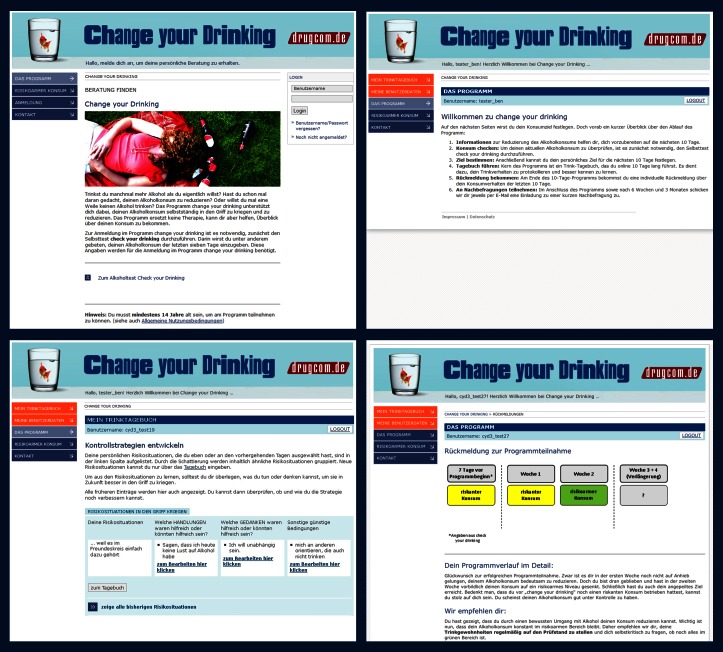
Screenshots of the Change Your Drinking intervention (version 2).

Moreover, 2 tailored and motivating feedbacks after 7 and 14 days were introduced in the intervention, thus extending the program’s length from 10 to 14 days. Both feedbacks address the participant’s current consumption levels, comparing those with the initial values (as reported in the Check Your Drinking self-assessment tool), and the previously chosen use-related goals. Tailored tips to cope with risk situations are also provided in the feedbacks. In addition, data entered in the diary is displayed graphically to give participants a quick look at changes in their alcohol use. Screenshots of version 2 of the intervention are shown in Figure 1.

### Statistical Analysis

The comparison of both program versions was conducted with generalized estimating equations (GEE) using Stata 11 (StataCorp LP, College Station, TX, USA), accounting for all 3 data collection points. The GEE analyses were modeled with unstructured correlation matrices between the data collection points. We assumed to have binary distributed data (binge drinking yes/no), Poisson-distributed data (alcohol use days and alcohol-related problems), and a negative binomial distribution with log link in the alcohol intake data (intake in grams).

In the GEE analyses, a group difference was assumed in case of a significant group × time interaction. To measure the development of both groups, the time effect of each outcome was examined. In case of any statistically significant result, Cohen’s *d* was calculated.

In a first step of data analysis, we tested whether group differences at baseline or the program usage (operationalized by online diary used: yes/no) moderated the effects of the group assignment on the study outcomes. In case of significance, the respective measure and its interaction with the group factor were included in the GEE; otherwise, it was not considered in the effectiveness testing.

For the intention-to-treat analyses (ITT), missing data was estimated by multiple imputations with Stata’s “ICE” command. We performed 10 imputations. The results from the multiple imputations were compared with completer analyses and last observation carried forward (LOCF) analyses. In the completer analyses, missing follow-up data was not imputed, so only those cases that provided follow-up data were analyzed. In the LOCF analyses, missing data was replaced with data from the preceding data collection point.

To compare both groups at study baseline and to determine whether baseline measures were predicting follow-up participation, logistic regression analyses were performed. In all analyses, we used a 2-sided significance level of alpha=.05. The study was powered to detect a group difference of *d*≥0.20. Therefore, we aimed for a sample size of N=624 (alpha=.05; power=0.80).

The research is reported in accordance with the E-CONSORT checklist [36] (see Multimedia Appendix 3).

## Results

### Flow of Participants

**Figure 2:**
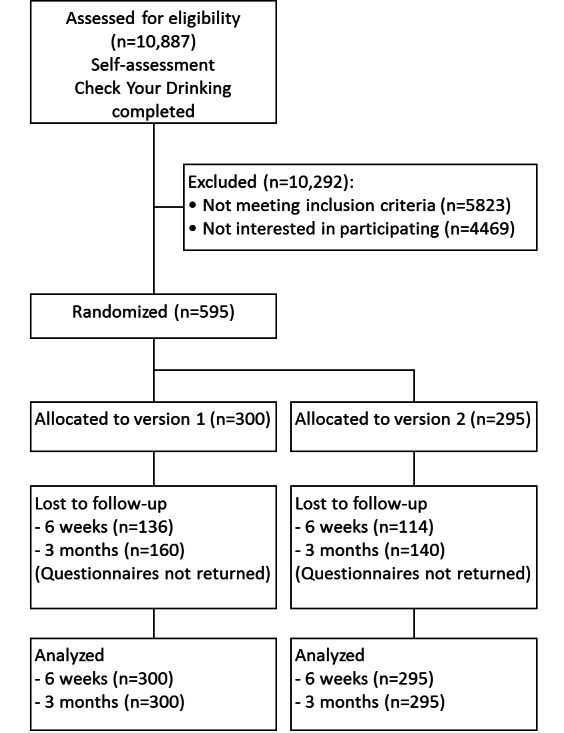
CONSORT flow diagram of participants.

Each time the Change Your Drinking starting page was opened, it was checked whether data for the Check Your Drinking self-assessment were available and whether the study criteria were fulfilled or not. In the trial period, Check Your Drinking was completed 10,887 times. A total of 5823 cases did not meet the study criteria and 4469 users refused to participate. Thus, 595 persons were included in the trial and were randomized, resulting in 2 approximately equally sized groups (see Figure 2). Participation in the trial (N=595) compared to refusing to participate (n=4469) was predicted by female gender (odds ratio [OR] 1.68, 95% CI 1.40-2.00, *P*<.001) and higher education (OR 1.39, 95% CI 1.22-1.58, *P*<.001). Thus, in the group of participants, females (38.8%) and individuals with high school education (65.0%) were more highly represented than in the group of nonparticipants (females: 27.4%, participants with high school education: 52.9%). Significant group differences were also found for the utilization of professional treatment (OR 1.83, 95% CI 1.10-3.03, *P*=.02) and age (OR 0.99, 95% CI 0.99-1.00, *P*=.04). That is, participants in the trial tended to use professional help more often (10.3% vs 5.9%) and were slightly younger (between-group *d*=0.09) than individuals who refrained from participating. Baseline alcohol use was not associated with trial participation (use days: OR 1.00, 95% CI 0.96-1.04, *P*=.95; intake in grams: OR 1.00, 95% CI 1.00-1.00, *P*=.16).

In total, 345 persons participated in the first follow-up and 295 in the second, resulting in follow-up rates of 58.0% and 49.6%, respectively. Although loss to follow-up was not predicted by group allocation (OR 0.82, 95% CI 0.56-1.20, *P*=.31), the level of education, program usage, and alcohol use were significant predictors. Thus, those who took part in the follow-up surveys were more highly educated (OR 1.36, 95% CI 1.14-1.61, *P*=.001), used the diary more often (OR 6.56, 95% CI 4.39-9.81, *P*<.001), and consumed less alcohol (OR 0.97, 95% CI 0.94-1.00, *P*=.04). However, we do not expect any significant bias on that account because these measures were all included in the equations of the multiple imputations.

### Sample Description

There were no significant group differences at baseline (see Table 2). Mean age of participants was approximately 30 years and most participants (364/595, 61.2%) were male. The education level of the participants was relatively high, with 387 of 595 (65.0%) having attended high school. A total of 534 of 595 (89.7%) of the participants were currently not using any other professional help to deal with their alcohol use.

In contrast to the baseline measures, the usage of the diary was significantly higher in the revised version of the program (OR 3.33, 95% CI 2.30-4.81; *P*<.001). According to the analysis plan, the diary usage was included in the first step of the effectiveness analyses.

### Effectiveness Results

Diary usage did not moderate the effects of the group assignment on any of the study outcomes (alcohol use days: beta = –0.04, 95% CI –0.22 to 0.14, *P*=.66; alcohol intake: beta = –0.01, 95% CI –0.13 to 0.11, *P*=.85; binge drinking: beta = 0.27, 95% CI –0.32 to 0.85, *P*=.37; alcohol-related problems: beta = 0.04, 95% CI –0.11 to 0.19, *P*=.56; risky drinking: beta = –0.49, 95% CI –1.02 to 0.03, *P*=.07); therefore, it was removed from the analysis.

There were no significant group differences in any of the primary outcomes of the study (see Table 3). Thus, the number of alcohol use days, the alcohol intake, the frequency of binge drinking, the number of use-related problems, and the indicators for risky consumption follow a similar direction in both groups.

However, significant overall reductions in alcohol use can be noted. Thus, after 3 months, participants in both program versions consumed on average 1.2 days (*d*=0.46) and 133.3 grams (*d*=0.59) less than at baseline. A significant reduction in alcohol-related problems (*d*=0.36), binge drinking incidents (reduction of 31.1%), and risky drinking (reduction of 23.6%) were seen. All the results were confirmed through completer and LOCF analyses.

**Table 2:** Participants’ characteristics at baseline and usage of the Change Your Drinking program (N=595).

Variable		Version 1 (n=300)	Version 2 (n=295)
**Gender, n (%)**			
	Male	177 (59.0)	187 (63.4)
	Female	123 (41.0)	108 (36.6)
Age, mean (SD)		29.8 (10.3)	29.0 (9.4)
Currently no professional help, n (%)		266 (88.7)	268 (90.8)
**Educational level, n (%)**			
	Basic school (Hauptschule)	25 (8.3)	23 (7.8)
	Middle school (Realschule)	57 (19.0)	64 (21.7)
	High school (Gymnasium)	203 (67.7)	184 (62.4)
	Other school	15 (5.0)	24 (8.1)
**Alcohol use**			
	Alcohol use days^a^, mean (SD)	4.1 (1.8)	4.3 (1.9)
	Alcohol intake^a^ (g), mean (SD)	313.4 (193.0)	318.5 (194.4)
	Binge drinking^a^, n (%)	278 (92.7)	271 (91.9)
	Alcohol-related problems, mean (SD)	2.4 (1.8)	2.3 (1.9)
**Usage of the program diary, n (%)**			
	Diary used at least once	167 (55.7)	238 (80.7)
	Diary used on all 10/14 days	127 (42.3)	163 (55.3)

^a^During the past 7 days.

**Table 3:** Effectiveness results^a^ of study primary outcomes.

Outcome	Version 1 (n=300)			Version 2 (n=295)			Group × time interaction^b^		Main effect of time	
	Baseline	6 weeks	3 months	Baseline	6 weeks	3 months	Beta (95% CI)	*P* value	Beta (95% CI)	*P* value
Alcohol use^c^ (days), mean (SD)	4.1 (1.8)	2.9 (3.1)	2.9 (4.1)	4.3 (1.9)	3.0 (2.9)	3.0 (2.6)	0.01 (–0.09, 0.11)	.88	–0.18 (–0.27, –0.08)	.002
Alcohol intake^c^ (grams), mean (SD)	313.4 (193.0)	162.2 (171.6)	183.3 (272.8)	318.5 (194.4)	151.8 (166.6)	181.9 (240.7)	–0.01 (–0.14, 0.11)	.84	–0.14 (–0.22, –0.07)	<.001
Binge drinking^c^ (yes), n (%)	278 (92.7)	190 (63.4)	194 (64.5)	271 (91.9)	178 (60.4)	171 (57.8)	–0.11 (–0.39, 0.17)	.44	–0.69 (–0.92, –0.47)	<.001
Alcohol-related problems^d^, mean (SD)	2.4 (1.8)	1.6 (2.1)	1.5 (3.2)	2.3 (1.9)	1.5 (1.9)	1.5 (2.3)	0.04 (–0.11, 0.19)	.56	–0.24 (–0.39, –0.09)	.004
Risky drinking^c^ (yes), n (%)	300 (100.0)	231 (77.1)	238 (79.3)	295 (100.0)	215 (72.9)	216 (73.4)	–0.10 (–0.47, 0.27)	.59	–0.64 (–0.92, –0.37)	<.001

^a^ITT analyses following multiple imputation. Results of complete case and LOCF analyses can be found in Multimedia Appendix 4.

^b^Comparison between version1 and 2 was conducted with the group × time interaction.

^c^During the past 7 days.

^d^During the past 30 days.

## Discussion

This study examined the effectiveness of the revised version of the fully automated alcohol intervention Change Your Drinking as compared to the original version of the program. The revised version lasts 14 days instead of 10 days, contains more feedback and interaction options, and is more strongly oriented toward the concept of relapse prevention [34,35]. However, in terms of drinking days, alcohol intake, and other use-related outcomes, the revised version did not yield superior results compared to the original version of the program. Instead, users of both versions reduced their use behavior in a similar way. For example, risky alcohol consumption was reduced in both groups by 23.6% after 3 months. Although trial participants differed from regular users of Change Your Drinking in terms of gender and education level, we assume that the results can largely be generalized to all users of the program.

The results suggest that the higher degree of interactivity, the improved feedback, the stronger emphasis on relapse prevention, and the program extension were not sufficient to significantly enhance the program effects. This is consistent with other findings that the effectiveness of a Web-based alcohol intervention is not increased by more feedback [17] or by the provision of a more elaborate and tailored intervention [37]. The findings are also reflected in the meta-analysis of Rooke and colleagues [10] on the effectiveness of computer-delivered alcohol and tobacco interventions. In this study, no association between the number of intervention sessions and the emphasis on relapse prevention and the intervention’s efficacy was found [10]. These results suggest that the effectiveness of self-help alcohol interventions can only be increased to a very limited extent by these features. To significantly enhance their effectiveness it might be necessary to include additional means of interaction, such as personal support by a counselor or therapist [14,38]. Moreover, it has to be noted that Web-based trials using an active comparison group (like ours) often yield very limited effects [10].

These results might question the time and costs spent developing elaborate automated programs. However, if an intervention is less sophisticated, it probably will be less attractive and, thus, be utilized by less people. This point is supported by the usage statistics of Change Your Drinking because the more elaborate version was used by more participants than the original version.

### Limitations

A key limitation to this study was the reliance on self-reported data. However, this type of data has repeatedly been shown to be reliable and valid [39,40]. Moreover, verification with urine samples or clinical interviews was not a practical option considering the widely scattered sample.

Because of the financial compensation given for follow-up participation, it cannot be ruled out that several participants tried to register more than once for the study. Although technical measures were taken to prevent this from happening, the anonymous study setting allowed a participant to sign up with different email addresses. However, we do not expect any bias in favor of any group due to this reason because multiple registrations (if any) presumably were equally distributed among both study groups.

Moreover, it should be noted that a significant number of participants did not take part in the final follow-up surveys. Therefore, we included all relevant participant data in the multiple imputations to estimate their follow-up data and crosschecked those results with completer analyses and LOCF analyses which came to very similar results.

To elucidate the effects of the interventions more thoroughly, it would have been desirable to collect additional data on their acceptability and usability. However, to keep the questionnaires short and the respondent burden as low as possible, we did not include these outcomes in this survey. A future evaluation study will deal with this topic in detail.

We did not include a no-intervention control group; therefore, we cannot determine the de facto effectiveness of the intervention. A comparison with other RCTs on Web-based alcohol interventions can provide only rough hints whether the reduction of alcohol use in the Change Your Drinking intervention exceeds mere trial participation. The overall reduction of alcohol intake (*d*=0.59) is considerably stronger than the effects of no-intervention control groups in other trials [20,41] (the difference of weekend drinking between baseline and 30-day follow-up in the trial of Doumas et al [20] and the difference of typical weekly drinking between baseline and 3-month follow-up in the trial of Cunningham et al [41], where effect sizes below *d*=0.10 were achieved). However, other RCTs on Web-based alcohol interventions show that nontreated groups can attain considerable performances up to *d*=0.40 (the difference of drinks last week between baseline and 3-month follow-up in the trial of Blankers et al [14] and the difference of alcohol intake in the past 7 days between baseline and 3-month follow-up in the trial of Jonas et al [42]). So these comparisons only suggest that both versions of Change Your Drinking to be somewhat effective, which leads to no clear conclusion. Hence, we cannot say whether the alcohol use reductions found in both groups are a consequence of the program participation, response bias, regression to the mean, or spontaneous remission. Nevertheless, since the original version of the intervention is freely accessible on the study website, a no-intervention control group was not feasible.

### Conclusion

The revised version of Change Your Drinking is not more effective in reducing alcohol use than the original version of the program. The extension of the program and its stronger elaboration presumably played a minor role in reducing alcohol consumption. However, analyses of the program usage suggest that it may be more attractive to participants. Acceptability and usability of both program versions will be examined in a future study.

## Multimedia Appendix 1

Information about the trial (German).

## Multimedia Appendix 2

Exemplary feedbacks of both program versions.

## Multimedia Appendix 3

CONSORT-EHEALTH checklist (V1.6.2) [36].

## Multimedia Appendix 4

Results of the complete case & LOCF analyses.

## Abbreviations

APS: Alcohol Problems Scale
AUDIT: Alcohol Use Disorders Identification Test
BZgA: Bundeszentrale für gesundheitliche Aufklärung (German Federal Centre for Health Education)
GEE: generalized estimating equations
ITT: intention-to-treat
LOCF: last observation carried forward
RCT: randomized controlled trial
SG: standard glasses
