# Supplementary material for: Two Fully Automated Web-Based Interventions for Risky Alcohol Use: Randomized Controlled Trial
Source: J Med Internet Res. 2013 Jun 6;15(6):e110. doi: 10.2196/jmir.2489 (PMC3720151; doi:10.2196/jmir.2489)
Supplement: Supplementary file 1 [file jmir_v15i6e110_app1.pdf]

drugcom.de

Information zur Studie im Rahmen von „change your drinking“

Das Programm „change your drinking“ unterstützt dich dabei, deinen Alkoholkonsum selbstständig in den Griff zu kriegen. Es beinhaltet ein Konsumtagebuch und gibt dir individuelle Rückmeldungen auf deinen Konsum. Die Dauer des Programms beträgt zwischen 10 und 14 Tagen und die Nutzung ist kostenlos.

Zurzeit führen wir eine **wissenschaftliche Untersuchung** zu „change your drinking“ durch. Im Rahmen der Studie geht es um die Frage, wie gut das Programm den Nutzerinnen und Nutzern hilft, ihren Alkoholkonsum zu reduzieren. Durch deine Teilnahme an der Studie kannst du uns helfen, diese Frage zu beantworten und „change your drinking“ zu verbessern!

Nach Programmende senden wir dir eine E-Mail zur Teilnahme an einer kurzen Nachbefragung zu. Für das vollständige Ausfüllen des Fragebogens senden wir dir einen **Warengutschein von Amazon.de in Höhe von 10 Euro** per E-Mail zu.

1. Was passiert in der Studie?

In der Studie wollen wir verschiedene Module des Programms testen. Das Ziel dieser Module ist es, Nutzerinnen und Nutzer noch individueller zu beraten.

2. Was bedeutet das für mich?

1) Du wirst einer von **zwei Studiengruppen** zugewiesen, die jeweils unterschiedliche Module erhält. Die Zuweisung erfolgt per Zufall. Sicher ist, dass du in jedem Falle eine kostenlose und wissenschaftlich fundierte Unterstützung bei der Reduzierung des Alkoholkonsums erhältst.

2) Wir bitten dich, an **einer kurzen schriftlichen Nachbefragung** teilzunehmen (Dauer ca. 5 Minuten), die direkt im Anschluss an das Programm erfolgt. Darin kannst du das Programm bewerten. Alle Daten werden anonymisiert ausgewertet (siehe auch 4.). Für einen vollständig und innerhalb von zwei Wochen nach Versanddatum ausgefüllten Fragebogen senden wir dir einen Warengutschein in Höhe von 10 Euro zu (einzulösen bei Amazon.de).

3. Du gehst keinerlei Verpflichtung ein

Du kannst dein Teilnahme an der Studie jederzeit und ohne Angabe von Gründen beenden. Wenn du nicht zu den Nachbefragungen eingeladen werden möchtest, schicke uns eine kurze E-Mail. Wir werden dann deine Kontaktdaten vollständig und unwiderruflich löschen.

4. Was passiert mit meinen Angaben, wenn ich an der Studie teilnehme?

- Für die Studie werden die Daten deiner Programmteilnahme sowie deine Angaben aus der Nachbefragung wissenschaftlich ausgewertet. Dabei werden nur anonymisierte Daten verwendet, so dass daraus generell kein Rückschluss auf deine Person möglich ist.
- Deine Angaben werden auf dem Server von „drugcom.de“ gespeichert. Ähnlich wie bei einer Online-Bank sorgen wir dafür, dass durch Verwendung hoher Sicherheitsstandards ein Zugriff Unbefugter weitestgehend ausgeschlossen ist. Alle deine Daten werden über eine verschlüsselte Verbindung (https) übermittelt.
- Darüber hinaus gelten die üblichen Datenschutzbestimmungen (siehe Impressum drugcom.de).

5. Noch Fragen zur Studie?

Für die Beantwortung deiner Fragen stehen wir dir gerne zur Verfügung. Unten findest du unsere Kontaktdaten. Grundsätzlich besteht nach Abschluss der Studie die Möglichkeit, eine Kurzzusammenfassung der Studienergebnisse zu erhalten. Schreibe uns hierzu einfach eine E-Mail.

6. Kontakt

Forschungsteam „change your drinking“
delphi Gesellschaft für Forschung, Beratung und Projektentwicklung
Ansprechpartner: Dipl.-Psych. Marc-Dennan Tensil
Kaiserdamm 8
14057 Berlin
Tel.: 030 - 39 40 97 85
E-Mail: changeyourdrinking@drugcom.de
