# Supplementary material for: Two Fully Automated Web-Based Interventions for Risky Alcohol Use: Randomized Controlled Trial
Source: J Med Internet Res. 2013 Jun 6;15(6):e110. doi: 10.2196/jmir.2489 (PMC3720151; doi:10.2196/jmir.2489)
Supplement: Supplementary file 2 [file jmir_v15i6e110_app2.pdf]

Version 1

Change your Drinking drugcom.de

Hallo, cyd3_test30! Herzlich Willkommen bei Change your Drinking ...

DAS PROGRAMM

Benutzername: cyd3_test30 LOGOUT

Rückmeldung zur Programmteilnahme

Du hattest vor, an maximal 0 Tagen insgesamt höchstens 0 Gläser Alkohol trinken. Nachdem was du im Trink-Tagebuch eingetragen hast, ist es dir nicht gelungen, diese Ziele zu erreichen.

Du hast häufiger und mehr Alkohol getrunken, als du geplant hattest. Zudem ergeben deine Tagebuchaufzeichnungen, dass du an mindestens einem Tag mehr als 4 Gläser Alkohol getrunken. Solltest du diese hintereinander getrunken haben, so hattest du vermutlich einen Alkoholrausch. Bedenke: Lebensgefährliche Alkoholvergiftungen sind nur eine der möglichen Folgen. Wer sich regelmäßig betrinkt, der hat z. B. mit einem schlechter werdenden Gedächtnis und verminderter Intelligenz zu rechnen (mehr dazu).

Es scheint dir schwer zu fallen, deinen Alkoholkonsum zu reduzieren. Wir wissen nicht, welche Gründe dabei in den letzten 10 Tagen eine Rolle gespielt haben. Vielleicht gab es etwas zu feiern, was mit Alkoholtrinken verbunden war. Vielleicht hast du aber auch gemerkt, dass der Drang, Alkohol zu trinken, stärker war, als der Wille, ihn zu reduzieren. Sollte letzteres auf dich zutreffen, dann nimm dieses Ergebnis als Anregung deinen Alkoholkonsum zu überdenken.

Empfehlung

Wichtig ist nun, dass du deinen Alkoholkonsum weiterhin beobachtest. Achte darauf, nicht mehr als 1 Glas Alkohol pro Tag zu trinken. Zum risikoarmen Alkoholkonsum gehören auch konsumfreie Tage. Erwachsene sollten an mindestens 2 Tagen pro Woche alkoholabstinent sein. Versuche, in Zukunft stärker darauf zu achten.

Vor allem solltest du es vermeiden, dich zu betrinken. Schau dir doch noch mal deine persönlichen Trinkstrategien an und überlege, welche davon geholfen haben oder welche vielleicht in Zukunft helfen könnten. Am besten ist, du druckst dir die Liste deiner Trinkstrategien aus und trägst sie in der nächsten Zeit immer bei dir. So kannst du dir deine Strategien schnell wieder in Erinnerung rufen, wenn du Alkohol trinkst.

Wir wünschen dir viel Erfolg dabei. Und solltest du einmal das Gefühl haben, erneut Unterstützung zu benötigen, so kannst du dich jederzeit wieder anmelden oder im drugcom-Chat eine Beraterin bzw. einen Berater ansprechen.

Deine Meinung ist gefragt: Wir würden gerne wissen, welchen Eindruck du von dem Programm change your drinking gewonnen hast, was dir gefallen hat und was nicht. Damit hilfst du uns, das Programm zu verbessern.

zum Feedback

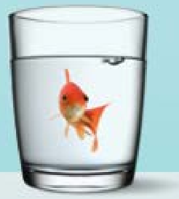

Rückmeldung zur Programmteilnahme

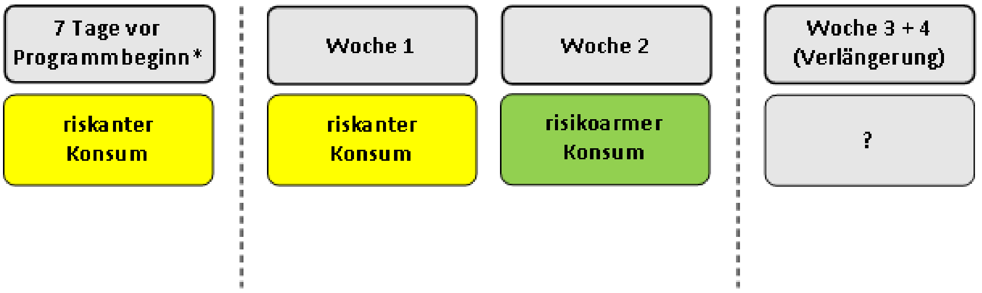

*Angaben aus check your drinking

Dein Programmverlauf im Detail:

Glückwunsch zur erfolgreichen Programmteilnahme. Zwar ist es dir in der ersten Woche noch nicht auf Anhieb gelungen, deinem Alkoholkonsum bedeutsam zu reduzieren. Doch du bist dran geblieben und hast in der zweiten Woche vorbildlich deinen Konsum auf ein risikoarmes Niveau gesenkt. Schließlich hast du auch dein angepeiltes Ziel erreicht. Bedenkt man, dass du vor „change your drinking“ noch einen riskanten Konsum betrieben hattest, kannst du stolz auf dich sein. Du scheinst deinen Alkoholkonsum gut unter Kontrolle zu haben.

Wir empfehlen dir:

Du hast gezeigt, dass du durch einen bewussten Umgang mit Alkohol deinen Konsum reduzieren kannst. Wichtig ist nun, dass dein Alkoholkonsum konstant im risikoarmen Bereich bleibt. Daher empfehlen wir dir, deine **Trinkgewohnheiten regelmäßig auf den Prüfstand zu stellen** und dich selbstkritisch zu fragen, ob noch alles im grünen Bereich ist.

Falls dein Konsum doch mal wieder zunehmen sollte, dann kannst du dich auf das besinnen, was du im Programm gelernt hast. Du kannst natürlich auch jederzeit wieder bei „change your drinking“ einsteigen und dich neu anmelden.

Tipps zum Umgang mit Risikosituationen

In der letzten Woche hast du Situationen in deinem Trinktagbuch markiert, in denen du zu viel Alkohol getrunken hast oder in denen es dir schwer gefallen ist, weniger zu trinken. Es ist daher wichtig, dass du dich mit diesen „Risikosituationen“ auseinandersetzt, um deinen Konsum in den Griff zu kriegen. Wir können dir hierzu ein paar Tipps geben:

- **In der vergangenen Woche ist es dir schwer gefallen, deinen Alkoholkonsum zu kontrollieren, weil du ein starkes Verlangen verspürt hast.**
  Es ist normal, dass das Verlangen nach Alkohol gerade in solchen Situationen oder unter solchen Umständen auftritt, in denen du früher getrunken hättest. Wenn du deinen Konsum langfristig kontrollieren möchtest wird es wichtig sein, dass du gerade dann nicht oder weniger trinkst, um deine bisherigen Konsummuster zu unterbrechen und zu verändern.

  Was kannst du tun?
  Es gibt verschiedene Möglichkeiten, dem Suchtdruck zu begegnen. Je häufiger du die Erfahrung machen kannst, dass du ihm nicht hilflos ausgeliefert bist, desto besser kannst du dich von einem „zwanghaften“ bzw. unkontrollierten Alkoholkonsum entfernen.

  Zunächst ist es wichtig, dass du dir klar machst, welche Auslöser es bei dir gibt, die Suchtdruck entstehen lassen. Dies sind häufig äußere Reize (z. B. bestimmte Orte oder Menschen) oder auch innere Befindlichkeiten (z.B. Gefühle wie Einsamkeit, Angst, Langeweile). Nimm dir einen Moment Zeit und überlege, in welchen Situationen du in der letzten Woche Suchtdruck hattest und schreibe sie dir auf. Wie kannst du Einfluss auf diese Auslöser nehmen? Inwieweit ist es dir zum Beispiel möglich, bestimmte Situationen zu vermeiden oder sie so zu verändern, dass der Suchtdruck nicht oder weniger stark auftreten wird. Du könntest dich zum Beispiel mit einem Freund

oder einer Freundin statt in einer Kneipe im Kino verabreden. Was kannst du tun, damit du weniger häufig Langeweile verspürst oder dich weniger einsam fühlst?

Es ist wichtig zu wissen, dass der Suchtdruck nur für eine begrenzte Zeitdauer anhält. In den allermeisten Fällen ist er nach maximal 20 Minuten wieder vorüber. Eine Strategie kann also darin bestehen, sich während dieser Zeitspanne abzulenken und mit anderen Dingen zu beschäftigen. Du könntest dir zum Beispiel etwas zu essen machen oder ein alkoholfreies Getränk zu dir nehmen. Verlasse den Ort oder die Situation, in der du dich gerade befindest und mach einen kleinen Spaziergang oder erledige ein Telefonat. Häufig ist es hilfreich, jemanden zu haben, den du in solchen Situationen anrufen kannst. Wer könnte dies sein?

Wenn du dein Trinkverhalten veränderst ist es sehr wahrscheinlich, dass du hin und wieder mit Verlangen konfrontiert wirst. Es ist also eher die Normalität als die Ausnahme. Vielleicht kann es dir helfen, wenn du dir in solchen Situationen vorsagst, dass es ein ganz normales Phänomen ist und kein Grund zur Sorge. Sage dir, dass das Verlangen auch wieder nachlassen wird und dass es dich stärkt, ihm nicht nachzugeben.

In der Regel sind positive Erwartungen an die Wirkung von Alkohol geknüpft. Hilfreich kann es deshalb sein, sich die negativen Konsequenzen vor Augen zu führen, die das Trinken in der Vergangenheit hatte. Du hattest schließlich gute Gründe dafür, etwas an deinem Konsumverhalten verändern zu wollen. Gerade dann, wenn du Suchtdruck erlebst, hast du die Möglichkeit, einen großen Schritt in Richtung einer Veränderung zu unternehmen.

- **Es ist dir in der vergangenen Woche schwergefallen, in Situationen, in denen du sonst Alkohol trinkst, um mehr Spaß zu haben und lockerer zu werden, deinen Alkoholkonsum zu kontrollieren.**
  Viele Menschen nutzen Alkohol, um Hemmungen zu überwinden. Dabei kann sich aber leicht ein Alkoholrausch entwickeln, der einen negativer Effekt nach sich zieht: Betrunkene Menschen benehmen sich oft plump oder gar aufdringlich und erscheinen weniger attraktiv.

  Was kannst du tun?
  Viele trinken Alkohol, weil sie glauben, dann mehr Spaß zu erleben. Das stimmt jedoch nur teilweise, denn sehr starker Alkoholkonsum hat häufig eine eher gegenteilige Wirkung. Ganz zu schweigen vom Kater am Tag danach!

  Überlege dir, wie du auch ohne Alkohol deine Hemmungen überwinden kannst. Auf andere zugehen und sich mit ihnen unterhalten ist etwas, was du lernen kannst. Nutze auch Alltagssituationen, um mit anderen zu reden. Sprich andere Menschen an, indem du z. B. nach der Zeit fragst oder dir sonstige Informationen einholst. Suche in der Schule oder an deinem Arbeitsplatz den Kontakt zu anderen, indem du dich zum Beispiel in den Pausen zu ihnen gesellst.

  Mach dir klar, was deine besonderen Fähigkeiten und Qualitäten sind! Je häufiger du dich überwindest, um einen Menschen anzusprechen, desto leichter wird es dir fallen, mit anderen in Kontakt zu kommen!

  Wenn du Alkohol eingesetzt hast, um dich zu belohnen, solltest du dir überlegen, wie du dich in Zukunft auf andere Art und Weise belohnen kannst. Womit kannst du dir eine Freude machen? Darüber hinaus gibt es zahlreiche Strategien, die dir dabei helfen können, die Trinkmenge zu begrenzen bzw. abstinent zu bleiben – auch auf Partys. Hier findest du Tipps und Infos.

Programmende oder Verlängerung?

Du hast das Programmende von „change your drinking“ erreicht. Aufgrund häufiger Nachfragen wurde die Möglichkeit eingerichtet, das Tagebuch noch weiter nutzen zu können. Es steht dir daher zwei weitere Wochen zur Verfügung, wenn du möchtest. Allerdings werden dir keine individuellen Feedbacks mehr gegeben. Statt einer ausführlichen Textrückmeldung erhältst du am Ende der Verlängerung ein kurzes Feedback über die Klassifizierung deines Alkoholkonsums der letzten 14 Tage.

Möchtest du verlängern?

- (•) Nein danke, ich beende das Programm jetzt.
- ( ) Ja, ich möchte das Tagebuch zwei weitere Wochen nutzen.

absenden
