# Supplementary material for: Two Fully Automated Web-Based Interventions for Risky Alcohol Use: Randomized Controlled Trial
Source: J Med Internet Res. 2013 Jun 6;15(6):e110. doi: 10.2196/jmir.2489 (PMC3720151; doi:10.2196/jmir.2489)
Supplement: Supplementary file 3 [file jmir_v15i6e110_app3.pdf]

CONSORT-EHEALTH Checklist V1.6.2 Report

(based on CONSORT-EHEALTH V1.6), available at [http://tinyurl.com/consort-ehealth-v1-6].

2489

Date completed

12/14/2012 10:18:57

by

Marc Tensil

Randomized controlled trial of two fully automated web-based interventions for risky alcohol use

TITLE

1a-i) Identify the mode of delivery in the title

Yes. "Randomised controlled trial of two fully automated web-based interventions for risky alcohol use"

1a-ii) Non-web-based components or important co-interventions in title

No non-web-based components or co-interventions were used in the study.

1a-iii) Primary condition or target group in the title

Yes. "Randomised controlled trial of two fully automated web-based interventions for risky alcohol use"

ABSTRACT

1b-i) Key features/functionalities/components of the intervention and comparator in the METHODS section of the ABSTRACT

Yes. ""Change Your Drinking" is a German-language diary-based, fully automated alcohol intervention. In 2010, a revised version of the program was developed. It is more strongly orientated to concepts of relapse prevention than the previous version, includes more feedback and offers more possibilities to interact with the program. Moreover, the program duration was extended from ten to fourteen days."

1b-ii) Level of human involvement in the METHODS section of the ABSTRACT

""Change Your Drinking" is a German-language diary-based, fully automated alcohol intervention."

1b-iii) Open vs. closed, web-based (self-assessment) vs. face-to-face assessments in the METHODS section of the ABSTRACT

Yes. "The effectiveness of both program versions was compared in a purely web-based open randomized controlled trial with follow-up surveys after six weeks and three months."

1b-iv) RESULTS section in abstract must contain use data

Yes, partly. "A total of 595 participants were included in the trial. Follow-up rates were 58 % after six weeks and 50 % after three months. No group-differences were found in any of the outcomes (P ≥ .44). However, the revised version was used by more participants (81 %) than the original version (56 %). A significant time-effect was detected in all outcomes."

1b-v) CONCLUSIONS/DISCUSSION in abstract for negative trials

Yes. "The duration and complexity of the programme played a minor role in reducing alcohol consumption. However, differences in the program usage suggest a higher attractiveness of the revised version."

INTRODUCTION

2a-i) Problem and the type of system/solution

Yes. "Despite high levels of problematic alcohol use, only few young people seek professional help. The World Health Organization (WHO) estimates that globally, 78 % of those in need for treatment remain untreated [9].

Internet-based self-help programs are developed to reduce this gap. Among others, their advantages include easy accessibility and relative anonymity. Online programs provide an appealing alternative especially to those who would abstain from face-to-face treatment due to fear of exposure or embarrassment. Meta-analyses show that online self-help programs have a rather small effect size, but due to their scalability they are a cost-effective way to reduce alcohol consumption in the population [10,11].

The German Federal Centre for Health Education (BZgA) is offering its free online self-help program "Change Your Drinking" for young adults since 2009. Designed to help users to reduce their alcohol consumption, while keeping the threshold for participation low, the program aims to give adolescents and young adults easier access to professional help."

2a-ii) Scientific background, rationale: What is known about the (type of) system

Yes. "Study results from the field of web-based tobacco interventions show that more frequent and intensive use of web content is related to higher levels of smoking cessation [12]. Therefore the program was revised and new modules designed to encourage reflection of one's own alcohol use were added. The revised program is more complex and more strongly orientated to concepts of relapse prevention. In addition, it includes more feedback and further possibilities to interact with the program. The duration of the program was extended from ten to fourteen days."

METHODS

3a) CONSORT: Description of trial design (such as parallel, factorial) including allocation ratio

Yes. "This paper examines whether the revised version of "Change Your Drinking" is more effective in reducing alcohol consumption than the original version. Outcomes were the alcohol use days, the total intake in grams, the occurrence of binge drinking (yes/no) and binge drinking (yes/no) (all referring to the last seven days prior to each survey) as well as the number of alcohol-related problems."

3b) CONSORT: Important changes to methods after trial commencement (such as eligibility criteria), with reasons

No changes were made during the trial.

3b-i) Bug fixes, Downtimes, Content Changes

No substantial technical issues had to be fixed. No content changes have been made.

4a) CONSORT: Eligibility criteria for participants

Yes. "In order to be invited for the study, participants had to be Internet literate, at least 18 years old, had to reach the cut-off of 8 points in AUDIT or had to consume more than 24/12 g (male/female) of pure alcohol per day on average in the past week."

4a-i) Computer / Internet literacy

Yes. "In order to be invited for the study, participants had to be Internet literate, at least 18 years old, had to reach the cut-off of 8 points in AUDIT or had to consume more than 24/12 g (male/female) of pure alcohol per day on average in the past week."

4a-ii) Open vs. closed, web-based vs. face-to-face assessments:

Yes. "The intervention and the study were conducted purely web based on the addiction prevention website www.drugcom.de and the alcohol prevention website www.kenn-dein-limit.de, both run by the BZgA (http://www.webcitation.org/6CstlbzBl). Recruitment of participants started in December 2010 and ended in March 2012. All users of the freely accessible "Check Your Drinking" self-assessment were invited for the study if they met the eligibility criteria described below."

4a-iii) Information giving during recruitment

Yes. Information given during recruitment is attached in the appendix.

4b) CONSORT: Settings and locations where the data were collected

Yes. "The intervention and the study were conducted purely web based on the addiction prevention website www.drugcom.de and the alcohol prevention website www.kenn-dein-limit.de, both run by the BZgA (http://www.webcitation.org/6CstlbzBl). Recruitment of participants started in December 2010 and ended in March 2012. All users of the freely accessible “Check Your Drinking” self-assessment were invited for the study if they met the eligibility criteria described below. The results of the self-assessment were used both for the “Change Your Drinking” program and as baseline data for the trial. "

4b-i) Report if outcomes were (self-)assessed through online questionnaires

Yes. "Trial data were collected via self-assessment in the baseline survey, as well as six weeks and three months afterwards."

4b-ii) Report how institutional affiliations are displayed

Yes. See the trial information file in the appendix.

5) CONSORT: Describe the interventions for each group with sufficient details to allow replication, including how and when they were actually administered

5-i) Mention names, credential, affiliations of the developers, sponsors, and owners

Yes. "Marc-Dennan Tensil and Benjamin Jonas are researchers at Delphi Gesellschaft, which developed “Change Your Drinking” on behalf of the Federal Centre for Health Education (BZgA). Evelin Strüber is a research consultant at the BZgA."

5-ii) Describe the history/development process

No formative evaluations were conducted previously.

5-iii) Revisions and updating

The intervention was not changed during the trial.It does not contain any dynamic components which change over time.

5-iv) Quality assurance methods

Intervention and trial were tested thoroughly during installation. We did not include this information in the paper as we found it to be trivial.

5-v) Ensure replicability by publishing the source code, and/or providing screenshots/screen-capture video, and/or providing flowcharts of the algorithms used

Yes. We included three screenshots of the trial and intervention.

5-vi) Digital preservation

Yes. "The intervention and the study were conducted purely web based on the addiction prevention website www.drugcom.de and the alcohol prevention website www.kenn-dein-limit.de, both run by the BZgA (http://www.webcitation.org/6CstlbzBl)."

5-vii) Access

Yes. "The intervention and the study were conducted purely web based on the addiction prevention website www.drugcom.de and the alcohol prevention website www.kenn-dein-limit.de, both run by the BZgA (http://www.webcitation.org/6CstlbzBl). Recruitment of participants started in December 2010 and ended in March 2012. All users of the freely accessible “Check Your Drinking” self-assessment were invited for the study if they met the eligibility criteria described below. The results of the self-assessment were used both for the “Change Your Drinking” program and as baseline data for the trial."

5-viii) Mode of delivery, features/functionalities/components of the intervention and comparator, and the theoretical framework

Yes. "Interventions

The internet-based self-help program “Change Your Drinking” is completely based on automated database-generated feedbacks.

Original version (Version 1)

“Change Your Drinking” is based on Solution Focused Brief Intervention and methods of Cognitive Behavioral Therapy. Berg and Millers [15] solution focused treatment approach concentrates on achieving concrete behavioral goals within relatively narrow timeframes. When participants start the “Change Your Drinking” program, they are to choose a use-related goal which must fall within the limits for low-risk consumption.

The participants are then given access to an online-diary to keep track on their alcohol use over the next ten days. Information on strategies to control alcohol use is also given. The program thus aims at developing self-awareness and self-regulatory skills [16,17].

On tenth day, participants receive feedback based on their alcohol use as reported in the online-diary. The feedback also refers on how well the use-related goal was met. The feedback is based on Miller and Rollnick’s principles of Motivational Interviewing [18].

Revised Version (Version 2)

In order to promote the confrontation with one’s own consumption pattern, the original version was revised and supplemented with new modules. Based on the principles of relapse prevention [19,20], participants are now asked on a daily basis to confront their risk situations and to develop or refine control strategies. Short and motivating feedbacks are provided to reinforce the reflection of one’s own alcohol use.

Moreover, two tailored and motivating feedbacks after seven and 14 days were introduced in the intervention, thus extending the programme’s length from ten to 14 days. Both feedback messages address the participant’s consumption levels with regard to their prior levels as reported, their levels in the past week and their chosen target levels. Tailored tips to cope with risk situations are provided. In addition, data entered in the diary is displayed graphically to give participants a quick look at how their consumption has changed."

5-ix) Describe use parameters

Yes. "When participants start the “Change Your Drinking” program, they are to choose a use-related goal which must fall within the limits for low-risk consumption.

The participants are then given access to an online-diary to keep track on their alcohol use over the next ten days."

5-x) Clarify the level of human involvement

Yes. "The internet-based self-help program “Change Your Drinking” is completely based on automated database-generated feedbacks."

5-xi) Report any prompts/reminders used

No reminders were sent to the users.

5-xii) Describe any co-interventions (incl. training/support)

There were no co-interventions or the like.

6a) CONSORT: Completely defined pre-specified primary and secondary outcome measures, including how and when they were assessed

Yes. "Trial data were collected via self-assessment in the baseline survey, as well as six weeks and three months afterwards. The past seven days were used as reference period for alcohol consumption. In order to quantify the alcohol intake, participants were first asked to indicate the number of alcohol use days in the previous seven days. Afterwards, they were requested to specify the number and type of alcoholic beverages being consumed on each drinking day. Using these details, the amount of pure alcohol and the number of standard glasses (SG) per day was calculated. A standard glass corresponds to approximately 9-13 grams of pure alcohol. If five or more SG were consumed on any of the previous seven days, this was classified as binge drinking.

Another outcome was risky consumption in the past seven days, as defined by the following factors: (1) an average of more than 24/12 g (male/female) of pure alcohol per day or (2) more than five days of consumption or (3) at least one incident of binge drinking in the reference period.

Alcohol-related problems were measures by a German version of McGee and Kypri’s Alcohol Problems Scale (APS) [13]. The scale consists of 14 items describing negative consequences of alcohol consumption such as vomiting, unprotected sexual intercourse or blackouts during the last 30 days. Each item is to be answered with “yes”, “no” or “no answer”.

The AUDIT was used as part of the baseline survey to test for the study criteria. A cut-off of 8 points as suggested by Babor et al. [14] was used to define risky alcohol consumption."

6a-i) Online questionnaires: describe if they were validated for online use and apply CHERRIES items to describe how the questionnaires were designed/deployed
Does not apply, as our main outcome was alcohol use.
6a-ii) Describe whether and how "use" (including intensity of use/dosage) was defined/measured/monitored
Yes. "As a measure of the program usage we tracked the diary-usage (used at least once: yes/no)."
6a-iii) Describe whether, how, and when qualitative feedback from participants was obtained
We did not obtain any qualitative feedback from the participants.
6b) CONSORT: Any changes to trial outcomes after the trial commenced, with reasons
There were no changes in trial outcomes after the trial commenced.
7a) CONSORT: How sample size was determined
7a-i) Describe whether and how expected attrition was taken into account when calculating the sample size
We did not take attrition into account, as we performed multiple imputations and thus could analyse the whole sample.
7b) CONSORT: When applicable, explanation of any interim analyses and stopping guidelines
Does not apply.
8a) CONSORT: Method used to generate the random allocation sequence
Yes. "...participants were randomly assigned either to the original (version 1) or the revised program (version 2) by a random number generator software."
8b) CONSORT: Type of randomisation; details of any restriction (such as blocking and block size)
There were no restrictions such as block randomization.
9) CONSORT: Mechanism used to implement the random allocation sequence (such as sequentially numbered containers), describing any steps taken to conceal the sequence until interventions were assigned
Yes. "...participants were randomly assigned either to the original (version 1) or the revised program (version 2) by a random number generator software."
10) CONSORT: Who generated the random allocation sequence, who enrolled participants, and who assigned participants to interventions
Yes. "...participants were randomly assigned either to the original (version 1) or the revised program (version 2) by a random number generator software."
11a) CONSORT: Blinding - If done, who was blinded after assignment to interventions (for example, participants, care providers, those assessing outcomes) and how
11a-i) Specify who was blinded, and who wasn't
Yes. "The participants were blind to the results of the randomization as they only received detailed information about the program version they were allocated to."
11a-ii) Discuss e.g., whether participants knew which intervention was the "intervention of interest" and which one was the "comparator"
The difference of both program versions was not outlined in the informed consent procedures. So participants were not able to tell whether they were allocated to the "intervention of interest" or the "comparator".
11b) CONSORT: If relevant, description of the similarity of interventions
Not applicable.
12a) CONSORT: Statistical methods used to compare groups for primary and secondary outcomes
Yes. "The comparison of both program versions was conducted with Generalized Estimating Equations (GEE) using Stata 11, accounting for all three data collection points. The GEE analyses were modeled with unstructured correlation matrices between the data collection points. We assumed to have binary distributed data (binge drinking yes/no), Poisson-distributed data (alcohol use days and alcohol-related problems) and normally-distributed data (alcohol intake in grams). In the measure of alcohol intake, statistical outliers were corrected beforehand, i.e. all values above a pre-defined limit (the third quartile plus 1.5 times the interquartile range) were set to this limiting value. This affected between four and five percent of cases at the different data collection points. In addition, this measure was root transformed to correct deviations from the normal distribution.
In the GEE analyses, a group difference was assumed in case of a significant group-by-time interaction. In order to measure the development of both groups, the time-effect of each outcome was examined. In case of any statistically significant result, Cohen's d was calculated.
In a first step of data analysis, we tested whether group differences at baseline or the program usage (operationalized by "online-diary used: yes/no") moderated the effects of the group assignment on the study outcomes. In case of significance, the respective measure and its interaction with the group factor were included in the GEE; otherwise, it was not considered in the effectiveness testing.
For the Intention-To-Treat analyses (ITT), missing data was estimated by multiple imputations with Stata's "ICE" command. We performed ten imputations. The results from the multiple imputations were compared with completer analyses and last-observation-carried-forward (LOCF) analyses. In the completer analyses, missing follow-up data was not imputed, so only those cases which provided follow-up data were analyzed. In the LOCF analyses, missing data was replaced with data of the preceding data collection point.
In order to compare both groups at study baseline and to determine whether baseline measures were predicting follow-up participation, logistic regression analyses were performed. In all analyses, we used a two-sided significance level of alpha=0.05. The study was powered to detect a group difference of d ≥ 0.20. Therefore, a sample of n=624 was aimed for (alpha=0.05; beta=0.20). "
12a-i) Imputation techniques to deal with attrition / missing values
Yes. "The comparison of both program versions was conducted with Generalized Estimating Equations (GEE) using Stata 11, accounting for all three data collection points. The GEE analyses were modeled with unstructured correlation matrices between the data collection points. We assumed to have binary distributed data (binge drinking yes/no), Poisson-distributed data (alcohol use days and alcohol-related problems) and normally-distributed data (alcohol intake in grams). In the measure of alcohol intake, statistical outliers were corrected beforehand, i.e. all values above a pre-defined limit (the third quartile plus 1.5 times the interquartile range) were set to this limiting value. This affected between four and five percent of cases at the different data collection points. In addition, this measure was root transformed to correct deviations from the normal distribution.
In the GEE analyses, a group difference was assumed in case of a significant group-by-time interaction. In order to measure the development of both groups, the time-effect of each outcome was examined. In case of any statistically significant result, Cohen's d was calculated.
In a first step of data analysis, we tested whether group differences at baseline or the program usage (operationalized by "online-diary used: yes/no") moderated the effects of the group assignment on the study outcomes. In case of significance, the respective measure and its interaction with the group factor were included in the GEE; otherwise, it was not considered in the effectiveness testing.
For the Intention-To-Treat analyses (ITT), missing data was estimated by multiple imputations with Stata's "ICE" command. We performed ten imputations. The results from the multiple imputations were compared with completer analyses and last-observation-carried-forward (LOCF) analyses. In the completer analyses, missing follow-up data was not imputed, so only those cases which provided follow-up data were analyzed. In the LOCF analyses, missing data was replaced with data of the preceding data collection point.
In order to compare both groups at study baseline and to determine whether baseline measures were predicting follow-up participation, logistic regression analyses were performed. In all analyses, we used a two-sided significance level of alpha=0.05. The study was powered to detect a group difference of d ≥ 0.20. Therefore, a sample of n=624 was aimed for (alpha=0.05; beta=0.20). "
12b) CONSORT: Methods for additional analyses, such as subgroup analyses and adjusted analyses

Yes. "The comparison of both program versions was conducted with Generalized Estimating Equations (GEE) using Stata 11, accounting for all three data collection points. The GEE analyses were modeled with unstructured correlation matrices between the data collection points. We assumed to have binary distributed data (binge drinking yes/no), Poisson-distributed data (alcohol use days and alcohol-related problems) and normally-distributed data (alcohol intake in grams). In the measure of alcohol intake, statistical outliers were corrected beforehand, i.e. all values above a pre-defined limit (the third quartile plus 1.5 times the interquartile range) were set to this limiting value. This affected between four and five percent of cases at the different data collection points. In addition, this measure was root transformed to correct deviations from the normal distribution.
In the GEE analyses, a group difference was assumed in case of a significant group-by-time interaction. In order to measure the development of both groups, the time-effect of each outcome was examined. In case of any statistically significant result, Cohen's d was calculated.
In a first step of data analysis, we tested whether group differences at baseline or the program usage (operationalized by "online-diary used: yes/no") moderated the effects of the group assignment on the study outcomes. In case of significance, the respective measure and its interaction with the group factor were included in the GEE; otherwise, it was not considered in the effectiveness testing.
For the Intention-To-Treat analyses (ITT), missing data was estimated by multiple imputations with Stata's "ICE" command. We performed ten imputations. The results from the multiple imputations were compared with completer analyses and last-observation-carried-forward (LOCF) analyses. In the completer analyses, missing follow-up data was not imputed, so only those cases which provided follow-up data were analyzed. In the LOCF analyses, missing data was replaced with data of the preceding data collection point.
In order to compare both groups at study baseline and to determine whether baseline measures were predicting follow-up participation, logistic regression analyses were performed. In all analyses, we used a two-sided significance level of alpha=0.05. The study was powered to detect a group difference of $d \geq 0.20$. Therefore, a sample of n=624 was aimed for (alpha=0.05; beta=0.20). "

RESULTS

13a) CONSORT: For each group, the numbers of participants who were randomly assigned, received intended treatment, and were analysed for the primary outcome

Yes. "Each time the "Change Your Drinking" starting page was opened, it was checked whether data for the "Check your Drinking" self-assessment was available and whether the study criteria were fulfilled or not. In the trial period, the "Check your Drinking" was completed 10,887 times. A total of 5,823 cases did not meet the study criteria and 4,469 users refused to participate. Thus, 595 persons were included in the trial and randomized, resulting in two approximately equally-sized groups (see Figure 1). In total, 345 persons participated in the first follow-up, 295 in the second, resulting in follow-up rates of 58 % respectively 50 %.
Though loss to follow-up was not predicted by the group allocation (OR=0.82; KI: 0.56-1.20; P=0.31), the level of education, the program usage and the alcohol use were significant predictors. Thus, those who took part in the follow-up surveys were more highly educated (OR=1.36; KI: 1.14-1.61; P=0. 001), used the diary more often (OR=6.56; KI: 4.39-9.81; P<0.001) and consumed less alcohol (OR=0.97; KI: 0.94-1.00; P=0.04). However, we do not expect any significant bias on that account, as these measures were all included in the equations of the multiple imputations."

13b) CONSORT: For each group, losses and exclusions after randomisation, together with reasons

Yes. "Each time the "Change Your Drinking" starting page was opened, it was checked whether data for the "Check your Drinking" self-assessment was available and whether the study criteria were fulfilled or not. In the trial period, the "Check your Drinking" was completed 10,887 times. A total of 5,823 cases did not meet the study criteria and 4,469 users refused to participate. Thus, 595 persons were included in the trial and randomized, resulting in two approximately equally-sized groups (see Figure 1). In total, 345 persons participated in the first follow-up, 295 in the second, resulting in follow-up rates of 58 % respectively 50 %.
Though loss to follow-up was not predicted by the group allocation (OR=0.82; KI: 0.56-1.20; P=0.31), the level of education, the program usage and the alcohol use were significant predictors. Thus, those who took part in the follow-up surveys were more highly educated (OR=1.36; KI: 1.14-1.61; P=0. 001), used the diary more often (OR=6.56; KI: 4.39-9.81; P<0.001) and consumed less alcohol (OR=0.97; KI: 0.94-1.00; P=0.04). However, we do not expect any significant bias on that account, as these measures were all included in the equations of the multiple imputations."

13b-i) Attrition diagram

We did not provide an attrition diagram.

14a) CONSORT: Dates defining the periods of recruitment and follow-up

Recruitment of participants started in December 2010 and ended in March 2012.

14a-i) Indicate if critical "secular events" fell into the study period

No critical "secular events" fell into the study period.

14b) CONSORT: Why the trial ended or was stopped (early)

The trial ended regularly.

15) CONSORT: A table showing baseline demographic and clinical characteristics for each group

Yes. "There were no significant group differences at baseline (see Table 1). Participants were approximately 30 years old, male participants were making up the majority. Educational level of the participants was relatively high, with over 60% attending grammar school or having reached A-Levels ("Abitur"). Roughly 90% of them were currently not using any other professional help to deal with their alcohol use."

15-i) Report demographics associated with digital divide issues

Yes. "There were no significant group differences at baseline (see Table 1). Participants were approximately 30 years old, male participants were making up the majority. Educational level of the participants was relatively high, with over 60% attending grammar school or having reached A-Levels ("Abitur"). Roughly 90% of them were currently not using any other professional help to deal with their alcohol use."

16a) CONSORT: For each group, number of participants (denominator) included in each analysis and whether the analysis was by original assigned groups

16-i) Report multiple "denominators" and provide definitions

Yes. See Table 2.

16-ii) Primary analysis should be intent-to-treat

Yes, primary analysis is ITT.

17a) CONSORT: For each primary and secondary outcome, results for each group, and the estimated effect size and its precision (such as 95% confidence interval)

Yes. See Table 2

17a-i) Presentation of process outcomes such as metrics of use and intensity of use

Our outcomes focused on alcohol use and not website usage etc.

17b) CONSORT: For binary outcomes, presentation of both absolute and relative effect sizes is recommended

Yes.

18) CONSORT: Results of any other analyses performed, including subgroup analyses and adjusted analyses, distinguishing pre-specified from exploratory

No subgroup analyses was done.

18-i) Subgroup analysis of comparing only users

No subgroup analyses was done.

19) CONSORT: All important harms or unintended effects in each group

We don't know of any privacy breaches or other harm to the participants due to intervention or trial participation. No major technial difficulties arose.

19-i) Include privacy breaches, technical problems

We don't know of any privacy breaches or other harm to the participants due to intervention or trial participation. No major technial difficulties arose.

19-ii) Include qualitative feedback from participants or observations from staff/researchers

No qualitative data was collected in course of the trial.

DISCUSSION

20) CONSORT: Trial limitations, addressing sources of potential bias, imprecision, multiplicity of analyses

20-i) Typical limitations in ehealth trials

Yes. "As in other web based RCTs [e.g. 26,27], a key methodical limitation was the reliance on self reported data. However, verification with urine samples or clinical interviews was not a practicable option considering the widely scattered sample.
Due to the financial compensation given for the follow-up participation, it cannot be ruled out that several participants tried to register more than once for the study. Although technical measures were taken to prevent this from happening, the anonymous study setting allowed a participant to sign up with different e-mail addresses. However, we do not expect any bias in favour of any group due to this reason, as multiple registrations (if any) presumably were equally distributed on both study groups.
Moreover, it should be noted that a significant number of participants did not take part in the final follow-up surveys. Therefore we included all relevant participant data in the multiple imputations to estimate their follow-up data and crosschecked those results with completer-analyses and LOCF-analyses which came to very similar results.
As we did not include a no-intervention control group we cannot determine the de facto effectiveness of the intervention. Hence, we cannot say whether the alcohol use reductions found in both groups are consequence of the program participation, response bias, regression to the mean or spontaneous remission. Nevertheless, since the original version of the intervention is freely accessible on the study website a no-intervention control group was not feasible."

21) CONSORT: Generalisability (external validity, applicability) of the trial findings

21-i) Generalizability to other populations

As eligibility criteria for the trial were very unrestrictive we expect a good generalizibility of the results.

21-ii) Discuss if there were elements in the RCT that would be different in a routine application setting

Except for the trial registration and the follow-up-questionnaires there are no differences between the intervention during the trial and the "Change-Your-Drinking"-intervention which now runs on the website www.drugcom.de

22) CONSORT: Interpretation consistent with results, balancing benefits and harms, and considering other relevant evidence

22-i) Restate study questions and summarize the answers suggested by the data, starting with primary outcomes and process outcomes (use)

Yes. "This study examined the effectiveness of the revised version of the fully automated alcohol intervention “Change Your Drinking” as compared to the original version of the program. The revised version lasts fourteen days instead of ten days, contains more feedback and interaction options, and is more strongly oriented towards the concept of relapse prevention [19,20]. However, in terms of drinking days, alcohol intake and other use-related outcomes, the revised version did not yield superior results compared to the original version of the program. Instead, users of both versions reduced their use behavior in a similar way. For example, risky alcohol consumption was reduced in both groups by 23.6% after three months."

22-ii) Highlight unanswered new questions, suggest future research

Yes. "As we did not include a no-intervention control group we cannot determine the de facto effectiveness of the intervention. Hence, we cannot say whether the alcohol use reductions found in both groups are consequence of the program participation, response bias, regression to the mean or spontaneous remission. Nevertheless, since the original version of the intervention is freely accessible on the study website a no-intervention control group was not feasible."

Other information

23) CONSORT: Registration number and name of trial registry

Yes. Trial registration: ISRCTN31586428; http://www.controlled-trials.com/ISRCTN31586428/ (Archived by WebCite at http://www.webcitation.org/6BFxApCUT)

24) CONSORT: Where the full trial protocol can be accessed, if available

No full trial protocol is available.

25) CONSORT: Sources of funding and other support (such as supply of drugs), role of funders

Yes. "ACKNOWLEDGEMENT
The study was funded by the Federal Centre for Health Education."

X26-i) Comment on ethics committee approval

Yes. "The study was approved by the ethics committee of the Department of Applied Human Sciences at the University of Magdeburg-Stendal (Ref. 4973-15) and was registered with Current Controlled Trials (http://www.controlled-trials.com/ISRCTN31586428/)."

x26-ii) Outline informed consent procedures

Yes. "Users who were willing to participate were then asked to register and to provide their informed consent by clicking an “I agree to participate”-button."

X26-iii) Safety and security procedures

On the study website, various options are communicated to get prefessional help.

X27-i) State the relation of the study team towards the system being evaluated

Yes. "CONFLICTS OF INTERESTS
Marc-Dennan Tensil and Benjamin Jonas are researchers at Delphi Gesellschaft, which developed “Change Your Drinking” on behalf of the Federal Centre for Health Education (BZgA). Evelin Strüber is a research consultant at the BZgA."
