# Supplementary material for: Two Fully Automated Web-Based Interventions for Risky Alcohol Use: Randomized Controlled Trial
Source: J Med Internet Res. 2013 Jun 6;15(6):e110. doi: 10.2196/jmir.2489 (PMC3720151; doi:10.2196/jmir.2489)
Supplement: Supplementary file 4 [file jmir_v15i6e110_app4.pdf]

Results of the complete-case- & LOCF-analyses

Table 2a: Effectiveness results (completer-only analyses)

	Version 1			Version 2			Comparison V1 / V2[a]		Main effect of time	
	Base-line (n=300)	6 weeks (n=164)	3 months (n=140)	Base-line (n=295)	6 weeks (n=181)	3 months (n=155)	Beta (95%-CI)	*P* value	Beta (95%-CI)	*P* value
**Alcohol use days[b]**	4.1 (1.8)	2.9 (1.9)	2.9 (1.8)	4.3 (1.9)	2.9 (2.0)	3.0 (2.1)	0.00 (-0.08; 0.07)	0.892	-0.19 (-0.23; -0.14)	<.001
**Alcohol intake (grams)[b]**	313.4 (193.0)	161.0 (139.7)	165.0 (140.4)	318.5 (194.4)	146.1 (125.8)	169.0 (155.3)	-0.04 (-0.16; 0.07)	0.483	-0.24 (-0.32; -0.15)	<.001
**Binge drinking: Yes[b]**	93%	62%	62%	92%	61%	58%	-0.07 (-0.35; 0.21)	0.617	-0.79 (-0.99; -0.59)	<.001
**Alcohol-related problems[c] (number)**	2.4 (1.8)	1.6 (1.8)	1.4 (1.6)	2.3 (1.9)	1.5 (1.6)	1.4 (1.6)	0.02 (-0.10; 0.15)	0.736	-0.25 (-0.34; -0.15)	<.001
**Risky drinking: Yes[b]**	100%	69%	71%	100%	67%	66%	-0.18 (-0.57; 0.20)	0.352	-0.73 (-1.02; -0.44)	<.001

[a]The comparison V1 / V2 was conducted with the group-by-time interaction.

[b]during the last seven days

[c]during the last 30 days

Values for use days, intake and alcohol-related problems denote the Mean and in brackets the Standard Deviation (SD); 95%-CI = 95%-Confidence Interval; V1=Version 1; V2=Version 2

Table 2b: Effectiveness results (LOCF-analyses)

	Version 1 (n=300)			Version 2 (n=295)			Comparison V1 / V2[a]		Main effect of time	
	Base-line	6 weeks	3 months	Base-line	6 weeks	3 months	Beta (95%-CI)	*P* value	Beta (95%-CI)	*P* value
**Alcohol use days[b]**	4.1 (1.8)	3.4 (2.0)	3.5 (1.9)	4.3 (1.9)	3.5 (2.1)	3.5 (2.2)	-0.02 (-0.05; 0.02)	0.447	-0.06 (-0.08; -0.03)	<.001
**Alcohol intake (grams) [b]**	313.4 (193.0)	238.8 (198.8)	245.8 (196.6)	318.5 (194.4)	221.8 (197.0)	234.4 (205.3)	-0.04 (-0.10; 0.01)	0.143	-0.10 (-0.13; -0.06)	<.001
**Binge drinking: Yes[b]**	93%	77%	78%	92%	72%	71%	-0.13 (-0.36; 0.09)	0.251	-0.33 (-0.49; -0.17)	<.001
**Alcohol-related problems[c]**	2.4 (1.8)	2.0 (1.9)	2.0 (1.8)	2.3 (1.9)	1.8 (1.8)	1.8 (1.8)	-0.02 (-0.08; 0.04)	0.505	-0.06 (-0.11; -0.02)	0.003
**Risky drinking:**	100%	83%	79%	100%	85%	78%	-0.22 (-0.63; 0.19)	0.285	-0.27 (-0.58; 0.05)	0.094

Yes[b]

[a]The comparison V1 / V2 was conducted with the group-by-time interaction.

[b]during the last seven days

[c]during the last 30 days

Values for use days, intake and alcohol-related problems denote the Mean and in brackets the Standard Deviation (SD); 95%-CI = 95%-Confidence Interval; V1=Version 1; V2=Version 2
